# Independent and Joint Effects of Prenatal Zinc and Vitamin A Deficiencies on Birthweight in Rural Sidama, Southern Ethiopia: Prospective Cohort Study

**DOI:** 10.1371/journal.pone.0050213

**Published:** 2012-12-13

**Authors:** Samson Gebremedhin, Fikre Enquselassie, Melaku Umeta

**Affiliations:** 1 College of Agriculture, Hawassa University, Hawassa, Ethiopia; 2 School of Public Health, Addis Ababa University, Addis Ababa, Ethiopia; 3 School of Medicine, Addis Ababa University, Addis Ababa, Ethiopia; Aga Khan University, Pakistan

## Abstract

**Background:**

The effects of prenatal Zinc Deficiency (ZD) and Vitamin A Deficiency (VAD) on birthweight are controversial and their interaction has not been investigated.

**Objective:**

To assess the independent and interaction effects of prenatal zinc and vitamin A deficiencies on birthweight in rural Sidama, Southern Ethiopia.

**Methodology:**

A community-based prospective cohort study design was employed. Six hundred fifty pregnant women in their second or third trimester were randomly selected and their serum zinc and retinol concentrations were determined. About 575 subjects were successfully followed until delivery and birthweight was measured within 72 hours after delivery. The association between the exposures and birthweight was examined using log-binomial and liner regression analyses. Potential interaction between ZD and VAD was examined using Synergy Index (SI).

**Results:**

The mean birthweight (± standard deviation) was 2896 g (±423). About 16.5% (95% CI: 13.5–19.6%) of the babies had Low Birthweight (LBW). Prenatal ZD and VAD were not significantly associated to LBW with Adjusted Relative Risk (ARR) of 1.25 (95 CI: 0.86–1.82) and 1.27 (95% CI: 0.86–1.87), respectively. Stratified analysis on the basis of gestational trimester showed that the occurrence of the deficiencies neither in the second nor third trimester were associated to LBW. The deficiencies did not show synergetic interaction in causing LBW [SI = 1.04 (95% CI: 0.17–6.28)]. Important risk factors of LBW were maternal illiteracy [RR = 1.80 (95% CI: 1.11–2.93)], female sex of the newborn [RR = 1.79 (95% CI: 1.19–2.67)], primiparity [RR = 1.16 (95% CI: 1.02–1.35)], short maternal stature [RR = 1.63 (95% CI: 1.06–2.51)] and maternal thinness [RR = 1.52 (95% CI: 1.03–2.25)]. In the linear regression model, elevated CRP was also negatively associated to birthweight.

**Conclusion:**

LBW is of public health significance in the locality. The study did not witness any independent or interaction effect of prenatal ZD and VAD on birthweight.

## Introduction

Low birthweight (LBW) is the single most important predictor neonatal survival and a significant determinant of post-neonatal infant mortality, childhood morbidity and cognitive development [1], [2]. The contemporary fetal programming hypothesis linked LBW with markedly increased risk of cardiovascular disease and diabetes in adult life [3]. Globally more than 20 million infants, approximately 15.5% of all births, are born with LBW [4].

In Ethiopia a handful of studies attempted to determine the prevalence of LBW. A community-based study in Southwestern Ethiopia found 10% prevalence [5] and health institution based studies conducted in various urban settings witnessed prevalences ranging from 8.6 to 15.4% [6]–[11]. The 2000 [12] and 2005 [13] demographic health surveys reported 8% and 14% prevalences based on mothers' reporting of birthweight; however the estimates may not be reliable due to recall and reporting errors.

Studies endeavored to evaluate the effect of prenatal zinc supplementation on birthweight concluded equivocally. A recent meta-analysis of Randomized Control Trials (RCTs) concluded that taking zinc during pregnancy does not prevent LBW but slightly reduces preterm birth [14]. Nevertheless, the finding is far from being conclusive as preterm birth is known to be the major risk factor of LBW. On the other hand, several observational studies reported positive association between maternal zinc status and birthweight [15]. However, the studies had limited sample size and did not adequately control confounders.

Pertaining vitamin A, a meta-analysis concluded that prenatal vitamin A supplementation does not significantly reduce risk of LBW [16]. Nonetheless, the analysis included only three RCTs. Based on observational design, studies in Israel [17], UK [18] and Guatemala [19] witnessed positive relation; whereas, studies in Bangladesh [20] and USA [21] found no association.

The purpose of the current study is to investigate the independent and interaction effects of prenatal zinc and vitamin A deficiencies on birthweight in rural Sidama, Southern Ethiopia. The study also assessed the prevalence and general determinants LBW. Despite the existence of many RCTs and observational studies which evaluated the effect of prenatal zinc and vitamin A status on birthweight, the current study is worth documentable as it has been conducted in area with high prevalence of zinc and vitamin A deficiencies. Further the potential interaction effect of the two deficiencies on birthweight had not been studied before.

The study was conducted based on cohort data of 575 pregnant women in the aforementioned locality. Earlier, another article [22] based on similar cohort had described the zinc status of the study subjects. Unlike the current article, the previous paper was principally focused on the magnitude and correlates of prenatal zinc deficiency.

## Methods

### Study design

This is a community-based prospective cohort study. Pregnant women were classified based on their prenatal serum zinc and vitamin A statuses and followed until birth. Birthweight was measured within 72 hours of birth.

### Study setting

The study was conducted from January to October 2011 in 18 randomly selected rural kebeles of Sidama zone, Southern Ethiopia. A kebele is the smallest administrative unit in Ethiopia comprising roughly 1000 households. In Sidama zone, of the total population nearing three million, 95% dwells in rural areas. The livelihood of 85% of the population depends on subsistent farming. Nearly half of the population lives in the midlands (1750 to 2300 m above sea level (ASL)); whereas 30% and 20% dwell in the highlands (>2300 m ASL) and lowlands (<1750 m ASL), respectively. On average a household in Sidama has 4.9 dwellers, 0.3 hectares of land and 0.5 heads of livestock. The detail description of the study area has been given elsewhere [22].

### Study subjects

**Figure 1:**
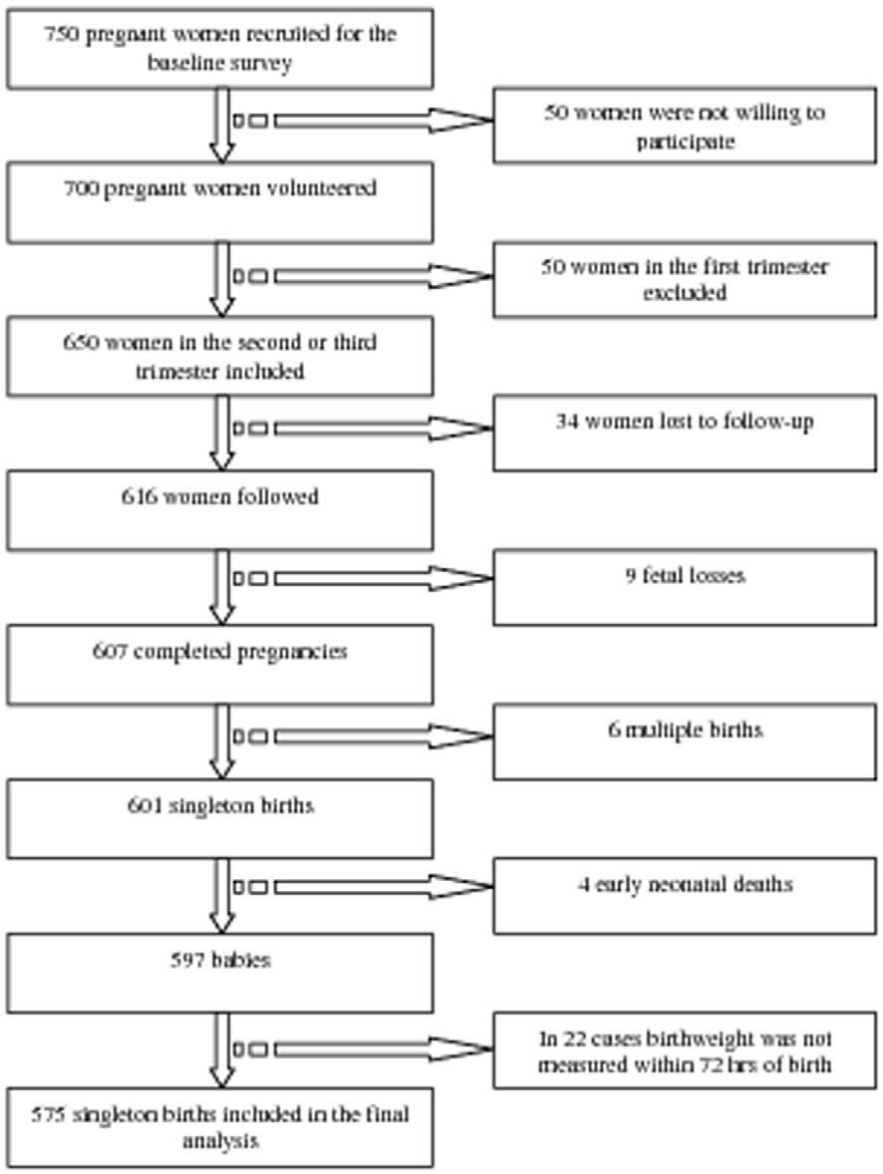
Flowchart of the cohort from exposure assessment to outcome ascertainment.

Pregnant women whose zinc and vitamin A statuses were determined at their second or third trimester and who later gave singleton live-births were eligible for the study. Babies visited after 72 hours of birth were excluded. Women whose exposure was determined in the first trimester were also excluded as the fetal weight gain in first trimester is known to be minimal. Flowchart of the cohort from exposure assessment to outcome ascertainment is given as follows (Figure 1).

### Sample size

The baseline study was designed to enroll 750 pregnant women. The basis of the sample size calculation has been given elsewhere [22]. Nevertheless, in the current study the adequacy of the available sample size for investigating the effects of the two exposures on LBW was assessed using double proportion sample size calculation formula. The power calculation was made using STATA/SE 11.0 with the inputs of 95% confidence level, 90% power and 1∶1 ratio between exposed and non-exposed subjects. Expected prevalences of LBW in exposed and non-exposed subjects were taken from prior studies [17], [23]. As a result, 97 vitamin A deficient and non-deficient, and 165 zinc deficient and non-deficient subjects would have been sufficient for the study. Hence the available sample size consisting of 228 vitamin A deficient and 347 non-deficient and 316 zinc deficient and 259 non-deficient subjects was judged to be adequate.

### Sampling technique

As described elsewhere [22], initially all the rural kebeles in the zone were listed and stratified into the three agro-ecological zones: lowlands, midlands and highlands. Then the total sample size (750 pregnant women) was divided to the three agro-ecological zones proportionally to their population size (20%, 50% and 30% respectively). From every stratum, 6 kebeles, a total of 18 kebeles, were selected using simple random sampling technique. Then sample size allotted for each stratum was distributed to the respective six kebeles proportionally to their population size. In every selected kebele house-to-house enumeration was conducted to identify pregnant women using presumptive symptoms of pregnancy (amenorrhea and/or increased uterine size) with subsequent pregnancy urine test (INSTANT- HCG®). Ultimately 750 subjects were selected using systematic random sampling technique. Nevertheless, for this specific paper, 575 pregnant women who were at their second or third trimester during the baseline survey were only considered.

### Data collection method

As described in the previous paper [22], in the baseline survey a structured and pretested questionnaire was used to assess the potential predictors of LBW. Apart from the main independent variables (prenatal vitamin A and zinc statuses), other independent variables of interest were maternal literacy (ability to read and write), maternal employment, household wealth index, agro-ecological zone, Antenatal Care (ANC) follow up, blood pressure during pregnancy, type of staple diet, distance from the nearby health facility, maternal thinness (maternal Mid-upper Arm Circumference (MUAC) less than 220 mm), maternal stunting (maternal height less than 145 cm), elevated CRP (CRP greater or equal to 5 mg/dl), newborn sex, maternal age and primiparity. The aforementioned variables were selected for the study based on review of literatures. Three trained and experienced data collectors gathered the baseline data at the nearby health posts using the local language. Maternal height and MUAC were measured to the nearest 0.1 cm using a stadiometer and MUAC tape following standard procedures.

The occurrence of births in the cohort were promptly identified and reported by prearranged local community health promoters and birthweight was measured within 72 hours of birth by trained health extension workers. Weight was measured to the nearest 100 g using calibrated Docbel BRAUN® scale.

### Blood sample collection and laboratory analysis

Blood samples were collected, stored, transported and processed following standard procedures. Six ml of blood was collected from antecubital vein using plain and closed SARSTEDT® blood collection system and stainless steel needles. The blood was allowed to clot for 20 minutes and centrifuged at 3000 rotations per minute for 10 minutes. Serum was extracted into screw-top vials within 40 minutes of sample collection. During this time few obviously hemolyzed samples were identified and discarded. In the entire process, the samples were protected from dust and direct light. In the field the samples were kept in icebox. The same day they were transported in icebox and kept frozen at −20°C until analyzed.

Serum zinc and retinol concentrations were determined at Ethiopian Health and Nutrition Research Institute using Varian SpectrAA® Flame Atomic Absorption Spectrometer and Shimadzu® High Performance Liquid Chromatography, respectively. C-Reactive Protein level (CRP) was determined qualitatively using latex HumaTex® kit.

### Data analysis

Data entered, screened and principally analyzed using SPSS 19.0. Additional analysis was made via STATA/SE 11.0. Independent t-test and one-way Analysis of Variance (ANOVA) were used to compare birthweight across categories of independent variables. Two of the major assumptions of ANOVA (homoscedasticity and normality of the dependent variable) were checked to be satisfied.

Wealth index was computed using Principal Component Analysis (PCA) as a composite indicator of living standard. A total of 23 variables related to ownership of selected household assets, size of agricultural land, quantity of livestock, materials used for housing construction, and ownership of improved water and sanitation facilities were considered for the analysis. Ultimately, seven principal components having eigenvalues greater than one were identified. Wealth index value was calculated by summing up the scores for the seven principal components. Ultimately, the five categories (poorest, poorer, middle, richer, and richest) were generated by splitting the wealth index values into 5 equal classes.

Log-binomial regression was used to control confounders and to model the risk LBW as function of multiple factors. In accordance with the framework of Kramer [1], the independent variables were entered separately in two blocks of indirect and direct factors. The indirect factors comprised agro-ecological zone and socio-demographic factors; whereas, the direct factors included ZD, VAD, maternal stunting, maternal thinness, CRP status, newborn sex, maternal age and primiparity. Only variables which showed significant association with LBW in simple regression models were included in the ultimate multivariate log-binomial models. The outputs of the model were given in Crude Relative Risk (CRR) and Adjusted Relative Risk (ARR). The goodness-of-fit assessed using Pearson chi-square and deviance tests.

Linear regression analysis was also used to control confounders and to assess the association between birthweight and various covariates. However, in this case only the direct factors were modeled as the r-squared value for indirect factors was found to be low. As the case of the log-binomial model, independent variables that turned out to be significant in bivariate linear models were considered for multivariate analysis. The major assumptions of the analysis (normality, homoscedasticity and independence error terms, linearity between dependent and independent variables and absence of multicollinearity) were not violated. The fitness of the multivariate model was assessed using the adjusted r-squared value.

Potential interaction between ZD and VAD in causing LBW was measured on additive scale. The additive scale is preferred as it is known to be correlated with biological interaction [24], [25]. The Synergy Index (SI) was calculated based on Rothman's formula. The 95% Confidence Interval (CI) for SI was computed as recommended by Hosmer and Lemeshow [26].

### Ethical Considerations

The study was conducted in confirmation of national and international ethical guidelines for biomedical research involving human subjects. Ethical clearance was secured from the institutional review board of Addis Ababa University. Informed written consent was taken from the study subjects. Nutrition education was given to all subjects and anemic women were given iron-folate supplementation.

## Results

### Socio-demographic information

Of 650 eligible pregnant women followed, 575 (88.5%) were included in the ultimate analysis. The exclusion was on the bases of loss to follow-up (34), birthweight measurement taken after 72 hours of birth (22), fetal loss (9), multiple birth (6) and early neonatal death (4). The retained and excluded subjects were not statistically different in terms of wealth index, agro-ecological zone, literacy, CRP status, maternal height, maternal MUAC, prenatal serum zinc and retinol concentrations *(P>0.05)* (Table 1).

**Table 1:** Comparability of retained and excluded subjects based on key socio-demographic and nutritional factors.

Key variables	Excluded subjects (n = 75)	Retained subjects (n = 575)	Test statistic and *P* values
Wealth index			*X^2^* = 1.053, *P* = 0.902
Poorest	17	112	
Poorer	12	105	
Middle	18	125	
Richer	13	119	
Richest	15	114	
Agro-ecological zone			*X^2^* = 2.606, *P* = 0.271
Lowlands	22	122	
Midlands	36	299	
Highlands	17	154	
Literacy			*X^2^* = 0.025, *P* = 0.874
Literate	26	194	
Illiterate	49	381	
CRP status			*X^2^* = 0.034, *P* = 0.854
CRP positive	7	50	
CRP negative	68	525	
Mean (± sd) maternal height (cm)	155.7±5.9	156.5±6.9	t = 0.893, *P* = 0.372
Mean (± sd) maternal MUAC (cm)	22.4±2.2	22.4±1.9	t = 0.296, *P* = 0.767
Mean (± sd) serum retinol (µmol/l)	0.88±0.38	0.82±0.42	t = 1.239, *P* = 0.216
Mean (± sd) serum zinc (µmol/l)	8.26±1.50	7.91±1.51	t = 1.926, *P* = 0.055

During the baseline survey 304 (52.9%) and 271 (47.1%) of the women were in their second and third gestational trimesters. Their mean age (± standard deviation) was 28.5 years (±5.4). Nearly half, 299 (52.0%), were from the midlands and the remaining 154 (26.8%) and 122 (21.2%) were from the highlands and lowlands, respectively. About two-third, 381 (66.3%), were illiterates and four-fifth, 462 (80.3%), were housewives. Among babies weighed, 289 (50.3%) were males.

### Prevalence and general determinants of LBW

The mean birthweight (± standard deviation) was 2896 g (±423). About 16.5% (95% CI: 13.5–19.6%) of the babies were born with LBW.

Maternal literacy showed affirmative influence on birthweight. Weight of babies born to literates (3023 g±428) was significantly higher than illiterates (2831 g±405) *(t = 5.276, P = 0.000)*. The risk of LBW was 1.80 (95% CI: 1.11–2.93) times higher in the earlier group. The mean birthweight increased from 2829 g in the poorest to 2951 g in the richest wealth quintiles. Yet the difference was not significant *(F = 1.899, P = 0.109)*. The prevalence of LBW in the five wealth quintiles were; poorest (23.2%), poorer (19.0%), middle (16.0%), richer (12.6%) and richest (12.3%). However in the log-binomial model the difference was not statistically significant. Likewise women involvement in Income Generating Activities (IGA) was not associated to LBW (Table 1).

On average, male babies weigh more than females by 103 g (95% CI: 34–172 g) *(t = 2.931, P = 0.004)* and females were 1.79 (95% CI: 1.19–2.67) times prone to LBW. The weight of babies born to primiparous women (2744 g±432) was significantly inferior to those born to parous women (2924 g±415) *(t = 3.76, P = 0.000)*. The risk was 1.16 (95% CI: 1.02–1.35) times elevated in the earlier group (Table 2).

**Table 2:** Output of the log-binomial regression analysis on general determinants of LBW in rural Sidama, Southern Ethiopia, Jan-Oct 2011.

Variables	Birthweight		Crude RR	Adjusted RR
	Low	Normal		
Women involvement in IGA				
Yes	6	69	1^r^	1^r^
No	89	411	2.23 (1.01–4.90)*	2.07 (0.94–4.55)
Literacy				
Literate	19	175	1^r^	1^r^
Illiterate	76	305	2.04 (1.27–3.27)*	1.80 (1.11–2.93)*
Wealth index quintiles				
Poorest	26	86	1.89 (1.04–3.43)*	1.52 (0.83–2.78)
Poorer	20	85	1.55 (0.83–2.91)	1.27 (0.68–2.41)
Middle	20	105	1.30 (0.69–2.46)	1.13 (0.60–2.13)
Richer	15	104	1.03 (0.52–2.03)	0.93 (0.47–1.83)
Richest	14	100	1^r^	1^r^
Two-way walking distance from the nearby health facility				
0–30 minutes	80	411	1^r^	-
Longer than 30 minutes	15	69	1.10 (0.66–1.81)	-
Staple diet				
Enset (Enset ventricosum) based	59	302	1^r^	-
Cereal based	36	178	1.03 (0.71–1.50)	-
Sex of the baby				
Male	32	256	1^r^	1^r^
Female	63	223	1.98 (1.34–2.94)*	1.79 (1.19–2.67)*
Parity				
Primipara	24	65	1.23 (1.07–1.40)*	1.16 (1.02–1.35)*
Parous	71	415	1^r^	1^r^
CRP status during pregnancy				
Negative	78	447	1^r^	1^r^
Positive	17	33	2.29 (1.48–3.54)*	1.26 (0.76–2.09)
MUAC				
≥220 mm	49	368	1^r^	1^r^
<220 mm	46	112	2.08 (1.45–2.99)*	1.52 (1.03–2.25)*
Maternal height				
≥145 cm	71	429	1^r^	1^r^
<145 cm	24	51	2.25 (1.52–3.34)*	1.63 (1.06–2.51) *
Maternal age				
15–24 years	21	106	1.09 (0.58–2.02)	-
25–34 years	60	296	1.11 (0.65–1.89)	-
35–49 years	14	78	1^r^	-
Agro-ecological zone				
Lowlands	14	108	0.87 (0.58–1.32)	-
Midlands	51	248	0.59 (0.32–1.06)	-
Highlands	30	124	1^r^	-
ANC during the pregnancy				
Yes	51	231	1^r^	-
No	44	249	0.83 (0.57–1.20)	-

The weight of babies born to women who had elevated CRP during pregnancy (2748 g±429) was significantly lower than their counterparts (2910 g±429) *(t = 2.601, P = 0.010)*. Yet in the multivariate log-binomial model the association was not significant (RR = 1.26 (95% CI: 0.76–2.09)) (Table 2).

Stunting (height <145 cm [27]) and thinness during pregnancy (MUAC<220 mm [28]) were strong predictors of birthweight. The birthweight of babies born to thin women (2697 g±361) was significantly inferior compared to their counterparts (2971 g±361) *(t = 7.255, P = 0.000)*. Likewise a 195 g (95% CI: 95–294 gm) significant difference was observed between babies born to non-stunted and stunted women. The risk of LBW was 1.63 (95% CI: 1.06–2.51) and 1.52 (95% CI: 1.03–2.25) times elevated in stunted and thin women, respectively (Table 2).

The risk of LBW was not significantly different across categories of maternal age, agro-ecological zone, ANC follow up, type of staple diet and distance from the nearby health facility (Table 2).

### Maternal zinc and vitamin A statuses and LBW

Standard cutoff points were applied to define prenatal ZD (serum zinc <7.6 µmol/l [29]) and VAD (serum retinol <0.7 µmol/l [30]). Using independent t-test analysis, statistically significant 82 g (95% CI: 9–150 g) and 76 g (95% CI: 6–145 g) decrease on birthweight was observed in babies born to women who had prenatal vitamin A and zinc deficiencies, respectively. In the bivariate log-binomial model, the deficiencies were also marginally associated with LBW. Nevertheless, in the multivariate model where adjustments were made for potential confounders (sex, parity, CRP status, thinness and stunting which were significantly associated with LBW in the bivariate analyses), VAD and ZD were not significantly associated to LBW with adjusted RR of 1.25 (95 CI: 0.86–1.82) and 1.27 (95% CI: 0.86–1.87), respectively (Table 3).

**Table 3:** Output of the log-binomial regression analysis on the association between LBW and Prenatal zinc and retinol statuses in rural Sidama, Southern Ethiopia, Jan–Oct 2011.

Variables	Birthweight		Crude RR	Adjusted RR
	Low	Normal		
Vitamin A status				
Normal	48	299	1^r^	1^r^
Deficient	47	181	1.49 (1.03–2.15)*	1.25 (0.86–1.82)
Zinc status				
Normal	34	225	1^r^	1^r^
Deficient	61	255	1.47 (1.01–2.16)*	1.27 (0.86–1.87)
Zinc-VA interaction				
Normal zinc and Normal VA	20	155	1^r^	1^r^
Zinc deficient and VA normal	28	144	1.42 (0.84–2.43)	1.30 (0.72–2.31)
VA deficient and zinc normal	14	70	1.46 (0.78–2.74)	1.31 (0.66–2.61)
Zinc deficient and VA deficient	33	111	2.01 (1.20–3.34)*	1.76 (1.00–3.11)

Previous studies witnessed the likelihood of trimester specific effects of micronutrient deficiencies on birthweight [31], [32]. In view of that, the log-binomial model was separately executed for women who were at their second and third gestational trimesters during the time of exposure assessment. The occurrence of ZD in the second or third trimester was not associated to LBW with RR of 1.31 (95% CI: 0.73–2.38) and 0.81 (95% CI: 0.46–1.40), respectively. Likewise, VAD in second or third trimester was not linked with LBW with RR of 1.26 (95% CI: 0.77–2.07) and 1.35 (95% CI: 0.71–2.57), respectively.

In order to investigate the possibility of synergetic interaction of the two deficiencies in causing LBW, the study subjects were categorized into four groups based on vitamin A and zinc deficiency statuses and analyzed accordingly (table 3). VAD and ZD did not show synergetic interaction with SI of 1.04 (95% CI: 0.17–6.28).

### Linear modeling of predictors of birthweight

In the bivariate linear regression model altitude of the kebeles, number of ANC visits, diastolic blood pressure during pregnancy were not associated to birthweight *(P>0.05)*. Hence, they were not considered for the multivariate analysis. Sex of the newborn, primiparity, maternal age, serum retinol, serum zinc, maternal height, MUAC and CRP status were exported to the multivariate model as they were significant in the bivariate analysis. In the ultimate model sex, primiparity, height, MUAC and CRP status were significant predictors of birthweight.

The final model explained 20.9% of the variability in birthweight (Table 4). A cm increment in maternal height and MUAC were associated with 11 g and 71 g increases in birthweight, respectively. Male babies were 102 g heavier than their counterparts. Parous mothers gave 129 g heavier babies compared to primiparas. Elevated CRP during pregnancy was linked to 180 g decline in birthweight. Serum zinc, retinol and maternal age were not significant predictors of birthweight.

**Table 4:** Output of the linear regression analysis on predictors of LBW in rural Sidama, Southern Ethiopia Jan–Oct 2011.

Variables	Unstandardized Coefficients		Standardized Coefficients	t	P
	Beta	SE	Beta		
Constant	−871.3	416.6		−2.092	0.037
Sex (0 = Female, 1 = Male)	101.7	31.8	−0.120	−3.194	0.001*
Nullyparous (0 = Primipara, 1 = Parous)	128.7	51.9	0.110	2.481	0.013*
Maternal age (years)	3.7	3.4	0.049	1.091	0.276
Serum zinc (µg/dl)	1.6	1.6	0.037	0.977	0.329
Serum retinol (µmol/l)	−0.1	1.4	−0.003	−0.086	0.932
Body height (cm)	10.8	2.4	0.172	4.458	0.000*
CRP (0 = Positive, 1 = Negative)	180.2	63.7	0.107	2.830	0.005*
MUAC (cm)	70.5	8.9	0.305	7.941	0.000*

* significant variables in the model.

## Discussion

LBW is considered as a problem great enough to trigger public health action when its incidence exceeds 15% [33]. Consequently, with the incidence of 16.5%, LBW requires focused intervention in the locality.

Due to the community-based nature of the study it was only possible to weigh newborns within 72 hours of birth. As birthweight is known to decline by 5–7% in the first three days of life [34], the study might have underestimated the birthweight with equivalent fraction.

In Ethiopia very few studies attempted to determine the prevalence of LBW. Most were conducted in major referral hospitals. The reported prevalence figures ranged from 8.6 to 15.4% [5]–[11]. The current study witnessed relatively higher prevalence probably due to the reason that it was conducted in rural area where the burden of the problem is expected to be high.

The study did not witness significant association between prenatal zinc status and infants' birthweight. The finding is in confirmation of the conclusion a meta-analysis that maternal zinc supplementation does not enhance birthweight [14]. However several observational studies conducted in the developed and developing world reported positive association between maternal zinc status and birthweight [15]. Nevertheless, nearly all of the studies reported the positive association merely based on correlation or t-test analyses. Among the studies, only few [15] applied multivariate analyses to control potential confounders. Accordingly the overwhelmingly positive association reported in the literatures can be due to the effect of confounding factors as most of the studies did not adequately control potential extraneous variables.

Observational studies on the relationship between maternal vitamin A and birthweight in apparently health subjects concluded divergently. A study in Israel reported that cord retinol along with gestational age explained more than a quarter of the variability of birthweight [17]. Studies in UK [18] and Guatemala [19] found a significant positive correlation and a study in India linked maternal night-blindness to LBW. However in the current study, parallel to studies conducted in Bangladesh [20] and USA [21], no significant association was witnessed. Systematic review of the available observational studies may help to resolve the existing conflicting findings.

In the current study the concurrent presence of VAD and ZD during pregnancy showed marginally insignificant effect on LBW (95% CI of 1.00–3.11). Nevertheless, it is important to interpret the finding in consideration of the fact that the sample size of the study was only calculated for the evaluation of the independent effect of the deficiencies on birthweight. Hence, in the measurement of the joint effects of the deficiencies on birthweight, the power of the study might have been compromised. Accordingly, in this area, further studies with optimal sample size are evidently required.

The fact that maternal zinc and retinol concentrations were measured only once during the entire pregnancy can potentially over or under estimate their association with birthweight as the exposure status to the deficiencies might not be fixed. Nevertheless, among women who were at their second gestational trimester during the baseline survey, follow-up survey conducted during their third trimester showed no significant change in their dietary diversity, food frequency and consumption of animal source foods. This might be taken as supporting evidence that their exposure status had not been assorted remarkably.

The linear regression model explained merely 20% of the variability in birthweight. This might have happened as some of the key predictors of low birthweight like weight gain during pregnancy, pre-pregnancy weight and malaria during pregnancy were missed from the model. A study showed that in the developing world the aforementioned factors can roughly explain 40% of the variability of birthweight [1].

Further, both in the linear and log binomial models, gestational duration has not been included as a covariate despite the fact that it is an important predictor of birthweight. This was due to the obvious difficulty of measuring gestational age in rural areas of developing countries where access to ultrasound is limited and estimation based the date of last menstrual period lacks accuracy. The exclusion of the variable from the regression analyses might have compromised the comprehensiveness of the models.

Several studies witnessed the positive contribution of superior household wealth status in reducing the burden of LBW [5], [35]. However, it was not the case in the current study. The unanticipated finding might have to do with the fact that wealth was quantified using a relative scale as measurement based on actual household income was not feasible. The relative scale might not be powerful when the wealth status of the population is reasonably homogenous.

In the current study male babies weigh more than females by about 100 gm and the risk of LBW was 1.8 times raised in females. Previous studies in Ethiopia and abroad concluded likewise [9], [11]. A systematic review concluded that on average, males weigh 150 g heavier than females [27].

Both in the log-binomial and linear regression models maternal MUAC was a strong correlate of birthweight. Especially in the linear model, removing the variable from the equation reduces the r-squared value from 21% to 12%. Even the study might have underestimated the association as pregnant women are commonly enrolled into supplementary and therapeutic feeding programs based on their MUAC. Studies in India [36], Brazil [37] and Bangladesh [38] also reported parallel findings.

Maternal height was also a strong predictor of birthweight. This is consistent to the understanding that in the developing world approximately 12% of low birthweight can be explained by maternal short stature [1]. Studies in India [35] and UK [39] concluded likewise. Though the exact mechanism how maternal height affects birthweight is not clear, it might be due to the mixed manifestation of genetic and intergenerational effect of malnutrition [27].

Unlike several studies that witnessed the beneficial effect of ANC in reducing the burden of LBW [9], [40], [41], the current study did not observe parallel findings. This might have to do with the inferior quality of ANC in the locality where only 42% and 76% of the ANC attendants received iron supplementation and nutrition education, respectively.

In the linear regression model elevated CRP during pregnancy was an important predictor of birthweight. The finding is in confirmation of the result of a systematic review that in the developing world approximately 4% of the burden of LBW is attributed to general illnesses during pregnancy and an extra 10% can be linked to malaria [1].

## Conclusion

The prevalence of low birthweight was 16.5%. LBW is of public health significance in the locality. Prenatal vitamin A and zinc deficiencies occurring in the second or third trimesters were not associated with increased risk of LBW. Similarly, the two deficiencies did not show synergetic interaction in causing LBW. Important risk factors of LBW were maternal thinness and stunting, primiparity, female sex of newborn and elevated CRP.
